# A three-arm randomized controlled trial of aerobic and resistance training in women with spinal cord injuries: Effects on physical fitness and pulmonary function

**DOI:** 10.1016/j.heliyon.2024.e32538

**Published:** 2024-06-25

**Authors:** Amir Hossein Haghighi, Atefeh Ahmadi, Roya Askari, Hadi Shahrabadi, Jeremy A. Moody, Joshua M. Miller, Filipe Clemente, Paulo Gentil

**Affiliations:** a Faculty of Sport Sciences, Hakim Sabzevari University, Sabzevar, 961797648, Iran; b Cardiff School of Sport and Health Science, Cardiff Metropolitan University, Cardiff, UK; c School of Physical Education and Sports, Nişantaşı University, Istanbul, Turkey; d Department of Kinesiology and Nutrition, University of Illinois, Chicago, IL, 60612, USA; e Escola Superior de Desporto e Lazer, Instituto Politécnico de Viana do Castelo, Viana do Castelo, 4900-347, Portugal; f Research Center in Sports Performance, Recreation, Innovation and Technology (SPRINT), Melgaço, 4960-320, Portugal; g Gdansk University of Physical Education and Sport, 80-336 Gdańsk, Poland; h College of Physical Education and Dance, Federal University of Goias, Goiânia, 74690-900, Brazil; i Hypertension League Federal University of Goias, Goiânia, 74605-050, Brazil

**Keywords:** Endurance training, Strength training, Lung function, Spinal cord injury, Resistance exercise

## Abstract

**Background:**

This study aimed to investigate the effects of different volumes of aerobic training (AT) and resistance training (RT) during a concurrent exercise training program on selected indicators of physical fitness and pulmonary function in women with spinal cord injury (SCI).

**Methods:**

Twenty-three inactive females with complete or incomplete SCI from T6 to L5 were divided into three groups: concurrent training with a focus on AT (CTAT; two weekly sessions of AT and one of RT), concurrent training with a focus on RT (CTRT; two weekly sessions of RT and one of AT), and control (CON). Tests were performed before and after an 8-week experimental period for indicators of pulmonary function, aerobic power, endurance performance, muscular strength and endurance, speed, and change of direction.

**Results:**

Markers of both aerobic and muscular fitness increased in the CTAT and CTRT groups, but not in CON. There were significant differences in aerobic power and endurance performance between the CTAT and CTRT groups, with greater changes in CTAT. Both CTAT and CTRT improved respiratory functions, with no differences between them (p > 0.05).

**Conclusions:**

CTAT and CTRT improved most of the indicators of physical fitness. However, CTAT should be used to achieve higher aerobic power and endurance without compromising muscle strength.

## 1 Introduction

Individuals with spinal cord injury (SCI) tend to have lower levels of physical activity, leading to decreased physical fitness [1,2]. This is associated with multiple risks such as cardiovascular disease, insulin resistance, osteopenia, and visceral obesity [3,4]. Respiratory dysfunction related to SCI is another important factor for morbidity and mortality [5,6] and is associated with an increased cost of breathing and inspiratory muscle fatigue [7]. Considering the potential importance of physical activity and fitness in managing the problems associated with SCI, it is important to promote health strategies and opportunities to increase regular physical activity in this group [8].

Many different exercise training programs are available to improve physical fitness and pulmonary function in individuals with SCI [9,10]. Studies with both resistance training (RT) and aerobic training (AT) have reported many benefits [[11], [12], [13]]. RT offers several benefits, such as increased muscle strength [13], which can be particularly helpful for those who have lost muscle mass due to inactivity or reduced mobility. Moreover, it can help individuals with SCI perform daily tasks such as wheelchair transfers and propulsion [14]. Additionally, RT can enhance cardiovascular health by reducing resting blood pressure and heart rate and increasing heart rate variability, which may lower the risk of cardiovascular disease [12,15]. RT can also improve metabolic function by increasing muscle mass and reducing body fat, which can help improve glucose metabolism and insulin sensitivity [16,17].

In contrast, AT can improve cardiovascular health by enhancing blood flow and oxygen delivery, thereby reducing the risk of heart disease [18,19]. AT can also increase endurance by improving the efficiency of the cardiovascular system, allowing individuals with SCI to perform daily activities and exercise for longer periods of time [14,20]. Finally, AT can improve respiratory function by increasing lung capacity and strengthening respiratory muscles, which can improve breathing and reduce the risk of respiratory infections [21,22].

RT and AT seem to act on different outcomes and through different pathways [10,11], which suggests that combining them in a concurrent training (CT) program might optimize the results [23]. However, following both the recommendations of two sessions of AT and two sessions of RT per week [2] might be too time-consuming and physically demanding for people with SCI. Therefore, it would be interesting to propose alternative protocols with reduced frequencies of AT or RT.

Based on this, the present study aimed to compare the effects of performing three weekly exercise sessions, with emphasis on AT (two sessions of AT and one of RT) or RT (two sessions of RT and one of AT), on physical fitness and pulmonary capacity of inactive people with SCI. Our hypothesis was that both protocols would promote benefits in muscle performance, aerobic capacity, and respiratory function, with no differences between them, and that the improvements in both protocols will be superior to the control group.

## 2 Methods

### 2.1 Study participants

Thirty-eight participants were invited and screened at a home care facility center for disabled people and 30 were included in the study, since eight did not meet the inclusion criteria. Study inclusion criteria involved being women aged between 25 and 60 years, complete or incomplete SCI between thoracic vertebrae 6 (T6) and lumbar vertebrae 5 (L5), use wheelchairs and other assistive devices such as walking frames, crutches, etc. for locomotion. ability to perform upper body training, not having visual and auditory limitations, being free of secondary health conditions (pressure sores, bladder infections, and cardiovascular diseases), being at least one-month post-injury, and had not participated in any regular exercise training in the past 6 months. The exclusion criteria was impossibility to complete the training protocol due to illnesses, pain, fatigue or personal reasons.

### 2.2 Study design and experimental approach

**Fig. 1:**
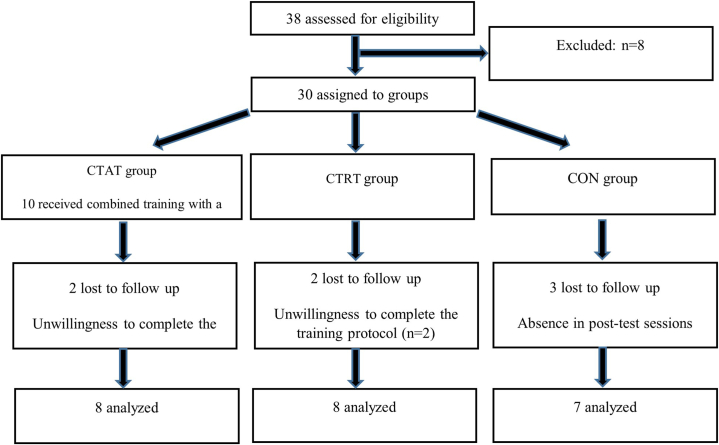
Flowchart of participant recruitment. CTAT: concurrent training with a focus on aerobic training; CTRT: concurrent training with a focus on resistance training; CON: control.

The current research adhered to the CONSORT (Consolidated Standards of Reporting Trials) guidelines for reporting randomized controlled studies Schulz et al., 2010.[24] This was an experimental study with random assignment and repeated measurements. The randomization sequence was generated electronically (http://www.randomization.com). Participants were randomly divided into three groups: concurrent training with a focus on aerobic training (CTAT, n = 10), concurrent training with a focus on resistance training (CTRT, n = 10), and control (CON, n = 10). Seven participants did not complete the study due to absence in the post-test sessions and unwillingness to complete the training protocol (two in CTAT, two in CTRT and three in CON). Therefore, final analysis were conducted on 23 participants (Fig. 1). Written informed consent was obtained from all participants. The study was approved by the relevant research ethics committee. The intervention period lasted for eight weeks. Before and after the intervention, the participants were evaluated for cardiovascular, muscular, and respiratory functions. The results were compared both with within and between groups.

### 2.3 Study procedures

Data were collected at the same time of day in weeks 0 (pre-intervention) and 8 (post-intervention) over three consecutive days. On the first day, demographic data (body mass, height, clinical assessment, etc.) and pulmonary function tests were collected. Physical fitness tests were performed on the second and third days. Day 2 included speed, change-of-direction, upper extremity muscle strength and endurance performance. Day 3 included upper extremity muscle endurance and aerobic power tests. All participants completed a familiarization session prior to data collection. Post-intervention tests were completed 48 h after the last training session.

### 2.4 Neurological examination and anthropometric measures

A trained neurologist determined the motor level and completeness of the injury through examination. Body mass was measured using a portable SECA scale (Seca770, Hamburg, Germany). Arm-span was measured with a steel tape (Rh-9050, Ronix, Iran). Participants were positioned with their back against the wall, with their arms abducted to the shoulder level while seated in their wheelchair. The measurement was taken as the distance between the left and right dactylion [25]. Body mass index (BMI) was estimated as body mass/arm span^2^.

### 2.5 Pulmonary function testing

Spirometry was based on American Thoracic Society standards [26] and was modified for individuals with SCI [27]. Pulmonary function was tested using a handheld spirometer (Spirolab III, MIR, Italy) while the participants were seated in their personal wheelchairs with the wheels locked. The participants completed three acceptable expiratory efforts, and the best values of FEV1 and FVC were analyzed and used to calculate the FEV1/FVC ratio. Peak expiratory flow (PEF) was measured using FVC [27].

### 2.6 Aerobic power test and endurance performance test

The 20-m shuttle test was completed by all participants to estimate the VO_2_max [28]. The participants were instructed to repeatedly propel themselves in their wheelchair between two lines 20-m apart until volitional fatigue. The intensity progressively increased, starting at a speed of 2.36 m/s, and increased by 0.14 m/s per minute. The test was completed when the subject failed to reach the 20-m line concurrent with the audio signal on two consecutive laps or due to volitional fatigue. VO_2_max was estimated based on Ramsbottom et al. [29].

The 12-min wheelchair push test (12WPT) was used to determine endurance [30]. The 12WPT was completed in an indoor 160-m track with markers every 40-m. Participants were told to push their wheelchair as far as possible for 12 min. The total distance was recorded upon the completion of the test. The participants were verbally encouraged throughout the test.

### 2.7 Muscular strength and endurance testing

Muscular strength was measured by estimating the bench press one-repetition maximum (1RM) load. The participants were then transferred from the wheelchair to a bench to perform the test. Once positioned properly on the bench, the participants lowered the barbell to the chest level and then returned to the starting position. Loads were adjusted so that participants could perform ≤10 repetitions. If the number of repetitions was >10, the load was increased and another attempt was performed after 5-min. The Brzycki equation was used to estimate the 1RM load [31,32]. Muscular endurance was measured by determining the number of repetitions performed in the bench press at 60 % 1RM [33].

### 2.8 Speed and change of direction test

**Fig. 2:**
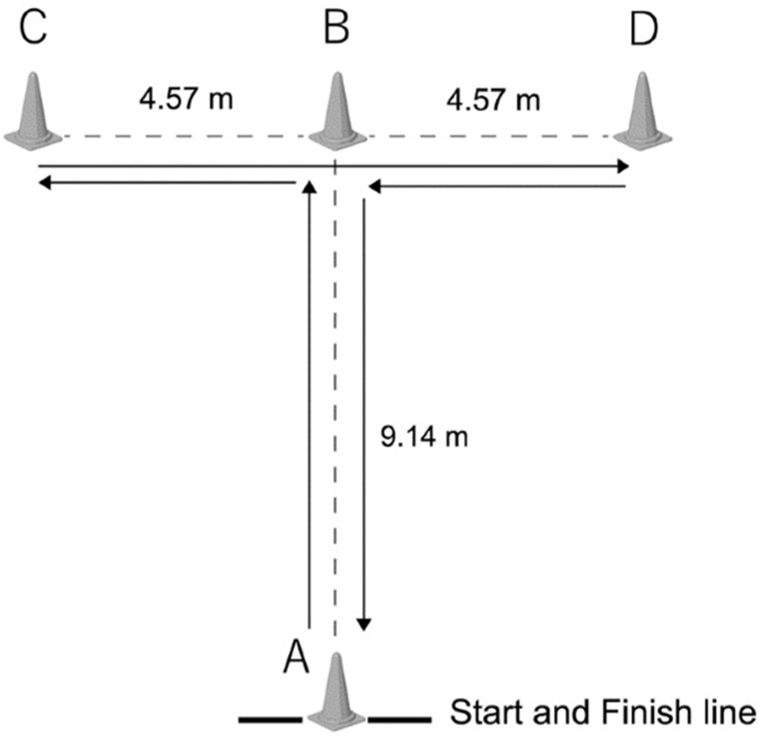
Schematic of the T-test agility test.

The participants completed two maximal speed tests over 20-m, with a 2-min rest interval. Time was measured using a stopwatch and the best time was used in the analysis [34]. Change-of-direction was measured using the T-test (Fig. 2). On ‘go,’ the participants propelled maximally to complete the test using self-selected turning directions [35]. All participants performed the test 2 times with at least a 3 min rest between trials, and the best time was used in the analysis.

### 2.9 Training sessions

Training was performed in a gym using adapted equipment. There were three familiarization sessions to confirm competency in how to perform the exercise program and to measure age-predicted maximum heart rate (APMHR) [35] and a six-repetition maximum test in the bench press test to determine training intensity.

CTAT and CTRT completed 8 weeks of training with 3 sessions per week (Table 1). Participants in the CTAT performed two sessions of AT (Wednesdays and Saturdays) and one session of RT per week (Mondays), while CTRT performed RT twice (Sundays and Thursdays) and AT once a week (Tuesdays). The CON group could participate in routine rehabilitation programs, but they could not participate in exercise training related to the protocol of the present study. Workload was monitored using a heart rate monitor (Medisana PM100, Germany). Each training session consisted of a 10-min warm-up, training (30–60-min duration based on individual ability), and a 10-min cool-down. AT consisted of wheelchair pushing and ball games. The RT involved bench press, rowing, overhead press, lateral raises, and combined movements with free weights.

**Table 1:** Concurrent training program protocol.

Exercise training type		Week 1	Week 2	Week 3	Week 4	Week 5	Week 6	Week 7	Week 8
RT Program	Intensity (% of 6RM)	70–80	70–80	70–80	70–80	70–80	70–80	70–80	70–80
	Sets × repetitions (n)	2 × 5	2 × 5	3 × 7	3 × 7	3 × 7	3 × 9	3 × 9	3 × 9
	Rest between sets (sec)	60	60	60	60	60	60	60	60
	Rest between exercises (sec)	90	90	90	90	90	90	90	90
AT Program	intensity (HR_max_)	70–80	70–80	70–80	70–80	70–80	70–80	70–80	70–80
	Repetitions (n)	2	2	2	3	3	3	4	4
	Exercise interval (min)	5	5	5	5	5	5	5	5
	Recovery interval (sec)	60	60	60	60	60	60	60	60

RT: resistance training; AT: aerobic training.

### 2.10 Statistical analysis

The sample size was estimated using G*Power software v3.1.9.4, with an alpha coefficient of 0.05, statistical power of 0.80, and effect size of 0.62, based on previous research [51], resulting in 23 participants overall. The within-subject reliability of physical fitness and pulmonary function tests was determined using ICC and with two different measures on participants of CTAT, CTRT and CON groups. Data normality was confirmed using the Schapiro–Wilk test. Paired t-tests were used for within-group comparisons and a within-group effect size (ES) of 0.20 was considered small, 0.50 medium, and 0.80 large [36]. Between-group comparisons were performed using analysis of covariance (ANCOVA), with baseline values as covariates. When necessary, multiple comparisons with confidence interval adjustment using the Bonferroni procedure were used for post-hoc comparisons. The between-group ES was calculated using partial eta squared (pη^2^) and 0.10 was considered small, 0.25 medium, and 0.40 large [36]. Data were analyzed using the IBM SPSS version 23 software (IBM SPSS Inc., USA). Statistical significance was set at p < 0.05.

## 3 Results

Intraclass correlation coefficient (ICC) were 0.93 (95 % CI 0.75, 0.98) for aerobic power, 0.96 (95 % CI 0.85, 0.99) for 12WPT, 0.94 (95 % CI 0.78, 0.98) for muscle strength 0.85 (95 % CI 0.50, 0.96) for muscle endurance, 0.98 (95 % CI 0.91, 0.99) for speed, 0.96 (95 % CI 0.87, 0.99) for change-of-direction, 0.95 (95 % CI 0.80, 0.99) for FEV1, 0.81 (95 % CI 0.40, 0.95) for PEFR, 0.94 (95 % CI 0.77, 0.99) FVC, 0.95 (95 % CI 0.83, 0.99) for VC and 0.97 (95 % CI 0.90, 0.99) for FEV1/FVC.

Of the 30 volunteers, 23 completed the study. Compliance with training was 90.1 ± 6.3 % for the CTAT group, and 91.7 ± 5.5 % for the CTRT group. The mean ± standard deviation of heart rate during AT was 132.2 ± 3.7 and 127.18 ± 6.6 bpm for CTAT and CTRT groups, respectively. The study population is shown in Table 2.

**Table 2:** Participants' demographic data.

		CTAT (n = 10)	CTRT (n = 10)	CON (n = 10)
Age (yr, mean ± SD)		35.9 ± 6.2	41.6 ± 13.6	32.3 ± 7.6
Arm Span (cm, mean ± SD)		155.1 ± 9.2	147.4 ± 8.0	148.6 ± 6.9
Body Mass (kg, mean ± SD)		68.7 ± 4.7	67.4 ± 9.3	61.4 ± 9.5
BMI (kg/m^2^, mean ± SD)		28.9 ± 4.1	31.0 ± 3.7	27.7 ± 3.0
Length of injury (yr, mean ± SD)		14.1 ± 10.2	16.8 ± 15.3	16.0 ± 7.8
Level of injury (n, %)	T6- T9	5 (50)	6 (60)	4 (40)
	T10-L1	3 (30)	3 (30)	4 (40)
	L2-L5	2 (20)	1 (10)	2 (20)
ASIA impairment scale (n, %)	A	1 (10)	2 (20)	1 (10)
	B	3 (30)	5 (50)	4 (40)
	C	4 (40)	2 (20)	3 (30)
	D	2 (20)	1 (10)	2 (20)

CTAT: concurrent training with a focus on aerobic training; CTRT: concurrent training with a focus on resistance training; CON: control; BMI: body mass index; yr: year; ASIA: American Spinal Injury Association.

### 3.1 Indicators of physical fitness

As shown in Table 3, both CTAT and CTRT significantly increased aerobic power, endurance performance, muscle strength, muscle endurance and speed (p=<0.001–0.025, d = 1.00–5.95, %Δ = 2.61–111.59). While CON reduced speed and change-of-direction (p = 0.010–0.015, d = 1.28–1.39, %Δ = 1.37–4.65).

**Table 3:** Pre-test and post-test comparison of physical fitness indices between the groups.

Variable	Groups	Pre- test	Post- test	% change	Paired sample *t*-test			ANCOVA		
					t	p-value	cohen's d	F	p-value	η^2^
Maximal aerobic power (ml^.^kg^.−1.^min^−1^)	CTAT	33.5 ± 4.0	34.9 ± 3.8	4.50	−8.77	c<0.001	3.10	17.76	c<0.001	0.65
	CTRT	30.0 ± 3.4	30.8 ± 3.5	2.61	−4.11	b0.004	1.45			
	CON	37.5 ± 2.2	37.5 ± 2.3	−0.22	0.93	0.388	0.35			
Endurance performance (m)	CTAT	532.5 ± 118.0	1103.8 ± 211.1	111.59	−9.90	c<0.001	3.50	33.60	c<0.001	0.78
	CTRT	352.5 ± 50.1	687.5 ± 102.5	97.79	−8.97	c<0.001	3.17			
	CON	400.0 ± 70.2	402.9 ± 82.0	0.47	−0.24	0.818	0.09			
Muscle strength of upper extremity (kg)	CTAT	11.1 ± 3.4	13.5 ± 5.7	17.50	−3.23	a0.014	1.14	6.31	b0.008	0.40
	CTRT	13.2 ± 5.0	17.7 ± 5.3	38.63	−16.84	c<0.001	5.95			
	CON	8.4 ± 1.3	8.7 ± 2.0	3.37	−0.51	0.631	0.19			
Muscle endurance of upper extremity (n)	CTAT	10.25 ± 4.1	14.0 ± 3.3	45.73	−5.35	b0.001	1.89	22.60	c<0.001	0.71
	CTRT	10.2 ± 2.3	14.9 ± 2.2	47.13	−14.28	c<0.001	5.05			
	CON	9.3 ± 1.7	9.4 ± 2.3	1.58	−0.26	0.805	0.10			
Speed (s)	CTAT	10.8 ± 0.8	10.1 ± 0.8	−6.61	4.34	b0.003	1.53	13.21	c<0.001	0.58
	CTRT	13.5 ± 1.3	12.3 ± 1.1	−7.92	2.83	a0.025	1.00			
	CON	14.1 ± 1.2	14.8 ± 1.4	4.65	−3.39	a0.015	1.28			
Change-of-direction (s)	CTAT	22.9 ± 0.9	22.2 ± 0.9	−2.96	2.53	a0.040	0.89	8.81	b0.002	0.48
	CTRT	28.5 ± 2.5	27.1 ± 1.9	−4.58	1.60	0.154	0.57			
	CON	30.6 ± 1.9	31.0 ± 1.8	1.37	−3.69	a0.010	1.39			

CTAT: concurrent training with a focus on aerobic training; CTRT: concurrent training with a focus on resistance training; CON: control; Values are presented as mean ± standard deviation.

a Significance level is p-value <0.05.

b Significance level is p-value <0.01.

c Significance level is p-value <0.001.

ANCOVA confirmed significant differences in aerobic power, endurance performance, muscle strength, muscle endurance, speed, and change-of-direction between the three groups (p=<0.001–0.008, η^2^ = 0.40–0.78). Post-hoc tests confirmed that the CTAT significantly improved aerobic power (p < 0.001, 95%CI = 0.78–2.15), endurance performance (p < 0.001, 95%CI = 368.17–758.78), muscle endurance (p < 0.001, 95%CI = 1.89–5.75), speed (p = 0.005, 95%CI = 0.62–3.84), and change-of-direction (p = 0.005, 95%CI = 1.35–8.35) more than CON, but this was not the case for muscle strength (p = 0.247, 95%CI = −0.63-3.56). CTRT significantly improved endurance performance (p < 0.001, 95%CI = 167.23–500.58), muscle strength (p = 0.007, 95%CI = 0.80–5.39), muscle endurance (p < 0.001, 95%CI = 2.77–6.63), speed (p < 0.001, 95%CI = 0.92–2.97), and change-of-direction (p = 0.003, 95%CI = 0.12–0.78) when compared to CON, but there was no difference for aerobic power (p = 0.119, 95%CI = −0.93-4.80). Changes in aerobic power (p = 0.019, 95%CI = 0.11–1.40) and endurance performance (p = 0.035, 95%CI = 14.05–445.08) were higher for CTAT than for CTRT. No other differences were found between the CTRT and CTAT groups (p > 0.05).

### 3.2 Pulmonary function testing

Pulmonary function indices measured using spirometry are presented in Table 4. Within group analysis showed significant changes for CTAT in PEFR, FVC and VC (p=<0.001–0.027, d = 0.99–3.71, %Δ = 7.88–12.17). For CTRT there were significant changes in FEV_1_, FVC and VC (p = 0.003–0.028, d = 0.98–1.60, %Δ = 13.09–14.29). The only significant change for the CON group was in FEV1 (p = 0.008, d = 1.48, %Δ = 3.69).

**Table 4:** Pre-test and post-test comparison of pulmonary function indices between the groups.

Variable	Groups	Pre-test	Post-test	% change	Paired sample *t*-test			ANCOVA		
					t	p-value	cohen's d	F	p-value	η^2^
FEV_1_ (l)	CTAT	2.49 ± 0.40	2.77 ± 0.32	13.00	−2.12	0.072	0.75	6.31	†0.008	0.40
	CTRT	1.86 ± 0.23	2.11 ± 0.27	14.29	−2.76	a0.028	0.98			
	CON	2.29 ± 0.27	2.21 ± 0.27	−3.69	3.93	b0.008	1.48			
PEFR (l/s)	CTAT	5.19 ± 0.45	5.57 ± 0.38	7.88	−3.54	b0.009	1.25	2.77	0.088	0.23
	CTRT	4.44 ± 1.32	5.00 ± 1.51	14.04	−2.02	0.083	0.71			
	CON	4.67 ± 1.23	4.34 ± 0.83	−3.41	0.65	0.540	0.25			
FVC (l)	CTAT	2.84 ± 0.42	3.15 ± 0.31	12.17	−2.79	a0.027	0.99	8.20	†0.003	0.46
	CTRT	2.07 ± 0.27	2.34 ± 0.33	13.27	−4.53	b0.003	1.60			
	CON	2.76 ± 0.38	2.67 ± 0.32	−2.83	1.75	0.131	0.66			
VC (l)	CTAT	2.67 ± 0.41	2.96 ± 0.39	11.07	−10.50	c<0.001	3.71	6.26	†0.008	0.40
	CTRT	1.93 ± 0.44	2.13 ± 0.29	13.09	−3.03	a0.019	1.07			
	CON	2.24 ± 0.45	2.25 ± 0.45	1.32	−0.12	0.906	0.05			
FEV_1_/FVC (%)	CTAT	87.68 ± 5.46	88.16 ± 9.41	0.54	−0.18	0.861	0.06	0.11	0.900	0.01
	CTRT	90.57 ± 11.42	91.64 ± 17.28	1.30	−0.22	0.832	0.08			
	CON	83.81 ± 9.56	83.09 ± 9.06	−0.69	0.40	0.701	0.15			

CTAT: concurrent training with a focus on aerobic training; CTRT: concurrent training with a focus on resistance training; CON: control; FEV_1_: forced expiratory volume in 1-s; PEFR: peak expiratory flow rate; FVC: forced vital capacity; VC: vital capacity; FEV1/FVC: forced expiratory volume in 1-s/forced vital capacity ratio; l: liters. Values are presented as mean (SD).

a Significance level is p-value <0.05.

b Significance level is p-value <0.01.

c Significance level is p-value <0.001.

The results of the ANCOVA analysis showed that there were significant differences in FEV_1_, FVC, and VC between the three groups (p = 0.003–0.008, η^2^ = 0.40–0.46), however there were no significant differences in PEFR, and FEV_1_/FVC (p = 0.088–0.900, η^2^ = 0.01–0.23). Post-hoc comparisons showed that FEV_1_ (p = 0.006, 95%CI = 0.12–0.78), FVC (p = 0.002, 95%CI = 0.15–0.69), and VC (p = 0.007, 95%CI = 0.10–0.66) changes were greater in the CTAT group than in the CON group. There were no differences between the CTRT and CON groups and intervention groups (CTAT and CTRT) (p > 0.05).

## 4 Discussion

The present research confirmed that CTAT and CTRT can improve several aspects of physical fitness in patients with SCI. However, CTAT seems to bring greater adaptations in aerobic power and endurance than CTRT, while CTRT did not show any advantage over CTAT. This suggests that one or two RT sessions per week results in similar benefits, which agrees with previous studies in trained and untrained young people without SCI [37,38]. However, although a weekly session of AT might be sufficient to improve cardiovascular fitness, the results are not optimal when compared with a higher frequency.

The CTAT group showed increases of 4.5 % (ES = 3.10) in VO_2max_, 111.6 % (ES = 3.50) in endurance performance, 17.5 % (ES = 1.14) in muscle strength, and 45.7 % (ES = 1.89) in muscle endurance after eight weeks of training. The CTRT group showed increases of 2.6 % (ES = 1.45) in VO_2max_, 97.8 % (ES = 3.17) in endurance performance, 38.6 % (ES = 5.95) in muscle strength, and 47.1 % (ES = 5.05) in muscle endurance. All these effects size are classified as large, according to Cohen [36]. In a previous study with two weekly sessions of RT and AT, Pelletier et al. reported an increase of 17.2 % in VO_2_max [23], while Kim et al. reported a 26 % increase after six weeks of AT performed three times a week [39]. The difference in aerobic capacity between these studies and the present study might be related to the characteristics of the participants, since participants in the present study had higher baseline VO_2_max and might be closer to their upper limits. In line with this, our results are similar to those of Hoekstra et al. [40] and Wouda et al. [41] who reported a 7–13 % increases in VO_2peak_ after 12–16 weeks of AT in subjects with SCI and VO_2peak_ averaging >30 ml. kg-1. min^−1^.

As for muscle strength, Pelletier et al. reported increases of 15.8 % [23] in bench press strength after performing two sessions of RT per week for 16 weeks; while Tørhaug et al. [42] reported increases of 16.4 % as a result of three weekly RT sessions for six weeks. Although the present results are considerably larger than these studies, they are similar to those of Duran et al., who reported a 46 % increase in 1RM bench press after 16 weeks of training performed three times a week [43]. The higher increases in the present study might be related to the test performed, since 1RM tests in complex exercises might be influenced by the effects of learning [44] and to the lower initial strength of the participants.

The CTAT and CTRT improved the speed (6.6 and 7.9 %, respectively) and change of direction (3.0 and 4.6 %, respectively) measured by the 20-m sprint and T-test. The ES for CTAT and CTRT were 1.53 and 1.0 for speed; and 0.89 and 0.57 for change of direction. Only the changes for change of direction in CTRT were classified as medium; being all other were considered large according to Cohen [36]. Presently, there is no research using CT to investigate these performance measures. In contrast, Ghasemnian et al. reported that RT performed three times a week was effective in improving speed in disabled female athletes, while agility was unchanged [45]. In contrast, Alves-Rodrigues et al. evaluated the effects of 12 weeks of functional strength training twice a week in people with SCI and reported significant increases in agility [52].

Few studies have attempted to investigate the effects of exercise training in pulmonary function in individuals with SCI and the results are conflicting [46], [47], [48],^50^]. Jung et al. reported that a land-based exercise program did not affect indicators of pulmonary function such as FVC, FEV_1_, and FEV1/FVC ratio [47]. However, Gaeini et al. showed that VC and FEV_1_ increased significantly after AT [46], which is in agreement with the findings of our study.

The present study reported improvement in the physical condition of SCI patients as a result of CT, with the difference that CTAT has a greater effect on the aerobic fitness of the patients than CTRT. It seems that three weekly exercise sessions lasting 30 to 60-min are sufficient to bring about many important improvements. Although physical activity has many proven benefits for individuals with SCI, many barriers must be overcome, as cited by William et al. [49]. For example, an increase in time and energy demand for activities of daily living may make it difficult to engage in an exercise program. Another important barrier is the perceived limited return compared to investment, since people with SCI might think that the benefits from exercise do not compensate for the time and energy costs. By looking at this, the present study seems to bring important elements for overcoming these barriers, as it reports important effects of a reduced-frequency exercise program in women with SCI. We hope the findings from this study will be useful for healthcare providers, public health officials, and researchers working to improve the health of patients.

Despite the estimation of the sample size based on previous studies, one of the main limitations of the research is the small sample size, which might lead to a risk of type II error and reduce the statistical power for analyzing smaller ES. This was mainly cause by the relative high dropout rate (seven out of 30 volunteers, which is >20 %). Other limitations include the lack of precise control of activities outside the training protocol and of the participants’ dietary habits. However, besides these limitations, the present findings may have important applications for health professionals and individuals with SCI.

## 5 Conclusion

The results of the current study confirm that a CT program brings important benefits for people with SCI, and that the performance of one session of either AT or RT still provides significant adaptations. However, performing one weekly session of RT and two sessions of AT might be preferred because it results in higher cardiorespiratory fitness without compromising muscle strength gains.

## Data availability

The data are available upon reasonable request.

## Funding statement

No external funding was received for this study.

## CRediT authorship contribution statement

**Amir Hossein Haghighi:** Project administration, Methodology, Investigation, Formal analysis, Data curation, Conceptualization. **Atefeh Ahmadi:** Investigation, Conceptualization. **Roya Askari:** Methodology, Investigation, Conceptualization. **Hadi Shahrabadi:** Methodology, Investigation, Conceptualization. **Jeremy A. Moody:** Writing – original draft, Visualization. **Joshua M. Miller:** Writing – original draft, Visualization. **Filipe Clemente:** Writing – review & editing, Writing – original draft, Visualization. **Paulo Gentil:** Writing – review & editing, Writing – original draft, Visualization, Formal analysis.

## Declaration of competing interest

The authors declare the following financial interests/personal relationships which may be considered as potential competing interests:Paulo Gentil is an Assistant Editor for Heliyon If there are other authors, they declare that they have no known competing financial interests or personal relationships that could have appeared to influence the work reported in this paper.
